# Concurrently Achieving 4.6 W/M^2^ and 120,000 Cyclability Enabled by Extendable Swing Arms in Rotational Triboelectric Nanogenerator

**DOI:** 10.1002/advs.75294

**Published:** 2026-04-14

**Authors:** Yihong Lin, Jiaming Zhou, In‐Yong Suh, Eunjong Kim, Kin Chiu Yip, Dae‐jin Kim, Jang‐Mook Jeong, Young‐Jun Kim, Jinyoung Jeon, Ju‐Hyuck Lee, Yoon‐Hwae Hwang, Sang‐Woo Kim, Dong‐Myeong Shin

**Affiliations:** ^1^ Department of Mechanical Engineering The University of Hong Kong Pokfulam Pokfulam Hong Kong 999077 China; ^2^ Department of Materials Science and Engineering Center for Human‐oriented Triboelectric Energy Harvesting Yonsei University Seoul 03722 Republic of Korea; ^3^ Department of Energy Science and Engineering Daegu Gyeongbuk Institute of Science and Technology (DGIST) Daegu 42988 Republic of Korea; ^4^ School of Transdisciplinary Engineering & BK FOUR Nanoconvergence Technology Division Pusan National University Busan 46241 Republic of Korea

## Abstract

Rotational triboelectric nanogenerators (r‐TENGs) are promising candidates for powering Internet of Things devices, owing to their ability to convert continuous mechanical motion into electricity. Nevertheless, improving their electrical output often comes at the cost of increased frictional degradation, which limits overall energy‐conversion efficiency and long‐term durability. Here, we introduce a novel r‐TENG to reconcile the intrinsic trade‐off between contact sufficiency and frictional dissipation. By inducing a hybrid kinematic profile, characterized by synchronized sliding and bouncing of fluorinated ethylene propylene blades, the device activates an auxiliary charge‐feeding mechanism while simultaneously mitigating wear. Experimental validation reveals that this configuration significantly enhances power density by 27% compared to constant‐length counterparts, achieving a root‐mean‐square voltage of ∼1.8 kV and a remarkable energy conversion figure of merit of 130.1 µC^2^ mN^−1^ m^−5^. Furthermore, we demonstrate the practical utility of this system through a self‐powered, indoor wind‐driven microbial disinfection platform. Utilizing the harvested energy to drive a Cu_3_P nanowire‐modified filter, the system achieves over 99.99% inactivation efficacy against both *Escherichia coli* and *Bacillus subtilis* via an irreversible electroporation mechanism. These findings underscore the potential of the extendable swing arm r‐TENG as a sustainable, dual‐function solution for ubiquitous energy harvesting and airborne pathogen control in indoor environments.

## Graphical abstract

A rotational energy harvesting device that successfully reconciles high power density with mechanical durability through a hybrid sliding‐and‐bouncing kinematic mechanism is reported. The success reported in our paper includes addressing a dilemma where enhanced electrical output comes at the cost of increased friction and wear, limiting the practical lifespan of the device.

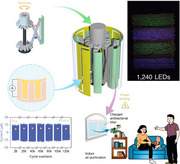

## 1 Introduction

The proliferation of Internet of Things (IoT) devices and pervasive distributed electronics places new demands on energy supply, characterized by massive spatial dispersion, low‐to‐moderate per‑node power requirements, and stringent reliability and maintenance constraints. Centralized supply architectures are ill‐suited to meet this modality at scale without prohibitive distribution infrastructure and recurring maintenance [1, 2, 3, 4, 5]. Consequently, ubiquitous decentralized renewable energy sources—coupled with local energy harvesting, small‑scale photovoltaics, micro‑wind, and hybrid storage systems—are increasingly receiving interest as a scalable and sustainable power supplier for distributed electronics [6, 7]. Recent studies highlight multiple advantages of embedding renewables at or near the point of consumption for IoT applications: reduced transmission losses, lower lifecycle carbon intensity, modular deployment enabling incremental scaling, and enhanced resilience against grid interruptions [6, 8, 9]. Moreover, energy harvesting techniques, including but not limited to triboelectric, piezoelectric, and photovoltaic, and low‑power electronics design can extend device lifetimes and reduce maintenance burdens, making local renewable provisioning technically attractive for many IoT use cases [10, 11].

Triboelectric nanogenerators (TENGs) have emerged as a transformative energy‐harvesting technology to power the distributed electronics, leveraging contact electrification and electrostatic induction to convert mechanical motion into electrical energy [12, 13, 14, 15, 16, 17, 18, 19, 20, 21, 22, 23, 24]. Among various TENG architectures, rotational designs are particularly promising due to their compatibility with industrial machinery, automotive systems, and flow energy applications. However, despite significant advancements in enhancing instantaneous electrical outputs [25, 26, 27, 28, 29, 30, 31]—such as open‐circuit voltage, short‐circuit current, and power density—achieving simultaneously high energy conversion efficiency remains a critical challenge. This dilemma stems from inherent trade‐offs: strategies that boost electrical performance often exacerbate mechanical and thermal losses, parasitic effects, or material degradation, ultimately reducing the net energy yield [32, 33, 34]. To maximize charge transfer, researchers have explored methods such as increasing contact force, enlarging interfacial area, and engineering micro/nanostructured surfaces [35, 36]. While these approaches enhance charge density, they also introduce frictional dissipation, adhesive hysteresis, and accelerated wear—factors that consume mechanical input energy and compromise device longevity.

Here, to reconcile these competing demands, we present a new class of extendable swing arms for a rotational TENG (r‐TENG), demonstrating high power output, superior energy‐conversion efficiency, and prolonged cycle life. The extendable swing arm imparts a combined sliding‐and‐bouncing motion to elastic blades, enabling an auxiliary charge‐feeding mechanism while diminishing frictional losses; together, these effects elevate both power density and energy conversion efficiency. Notably, this design achieves a 27% enhancement in power density, a peak RMS voltage of ∼1.8 kV, an impressive energy conversion figure of merit of 130.1 µC^2^ mN^−^^1^ m^−^^5^, and outstanding stability over 120,000 continuous cycles. Key design parameters governing electrical output are systematically optimized and experimentally validated, and the capability of r‐TENGs to harvest ambient wind energy is demonstrated. Furthermore, the practical utility of this system is exemplified by its successful deployment in a self‐powered microbial disinfection platform driven by indoor airflow, which displays over 99.99% disinfection efficiency against both *Escherichia coli* and *Bacillus subtilis*. Given sufficient electrical power delivery, reduced frictional dissipation, and longevity, our findings indicate that our r‐TENG with extendable swing arms is a promising route toward sustainable, widespread electricity sources for driving a broad spectrum of distributed electronic devices.

## 2 Results and Discussion

### 2.1 Overview of Structure Design

**FIGURE 1:**
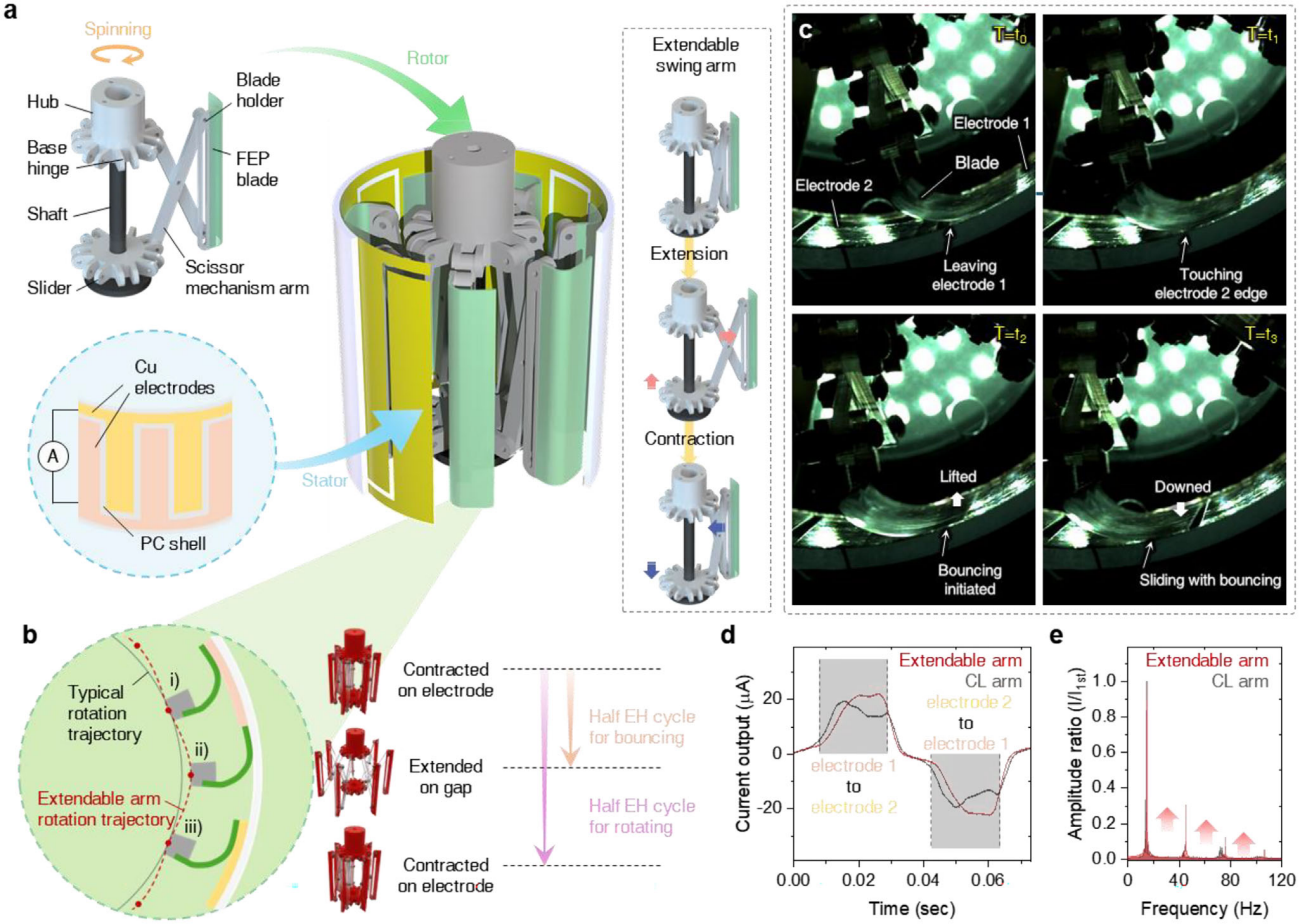
A rotational triboelectric nanogenerator featuring extendable swing arms. (a) Illustration and design explosion diagram of the r‐TENG equipped with extendable swing arms, enabled by a scissor mechanism. (b) Schematic diagram of the distinguished sliding and bouncing mechanism in the single swing arm. (c) Snapshot images of the sliding and bouncing motions in the extendable swing arm, captured by a high‐speed camera. (d) The current waveforms of r‐TENGs with extendable (red) and CL (gray) swing arms at a rotational speed of 124 rpm. (e) Fourier‐transformed current amplitudes, normalized by the peak amplitude of the rotation frequency, *f_r_* = (124 *rpm* × 7 *blades*)/60 *min* = 14.5 *Hz*.

The device schematic of a rotor [37] with extendable swing arms is shown in Figure 1a, which is mainly composed of a base hinge, a slider, and seven extendable swing arms made of a scissors mechanism and an elastic fluorinated ethylene propylene (FEP) blade. FEP was selected as the negative triboelectric material because of its strong electron affinity and highly negative position in the triboelectric series [38], which favors efficient charge generation. Compared with other commonly used negative triboelectric materials, such as polydimethylsiloxane (PDMS) and silicone rubber, FEP also exhibits a lower friction coefficient and superior mechanical durability, making it more suitable for repeated sliding contact under continuous rotational operation. Structurally, the scissors mechanism arms are extendable with rotation speeds, reaching up to 111.3 mm at 200 rpm (Figure S1). The stator consists of a shaft and a cylinder shell, and seven sets of interdigitated copper electrodes are attached to the inner surface of the shell. The design principle of the electrodes lies in securing the synchronized blade contact and separation over the course of rotation. The holes, located at the center of the base hinge and the slider in the rotor, are designed to fit into the shaft of the stator so that the rotor and stator are connected to construct a rotational triboelectric nanogenerator (r‐TENG) by inserting the rotor into the shaft. When the rotor starts to rotate, the elastic blades sweep the inner surface of the stator shell. As the length of the extendable swing arm is readily adjustable, the rotation trajectory relies on the depth profile of the stator inner wall instead of following the typical rotation trajectory, leading to the sliding and bouncing motion of the elastic blades (Figure 1b). It should be noted that the bouncing happens in every electrode gap, while the sliding over the single set of electrodes engenders a single cycle of rotational energy harvesting, resulting in the bouncing frequency being twice that of rotational energy harvesting.

The snapshots of rotating extendable swing arms confirmed that the obvious bouncing of elastic blades on the electrodes has been found (Figure 1c and Movie S1), leading to the wavy trajectory of the blade holder, while the elastic blades affixed to a rotating arm of constant length (CL) show no noticeable bouncing (Figure S2 and Movie S2). The current output from the r‐TENG shows that the plateau at the maximum current value lasts longer than the one where bouncing and sliding motions are not involved (Figure 1d). The fast Fourier transform analysis reveals that the higher‐order peaks were significantly enhanced in the extendable arm rotation (Figure 1e), further corroborating that the bouncing occurs while the elastic blades slide over the stator wall.

### 2.2 Output Performance and Working Principle

**FIGURE 2:**
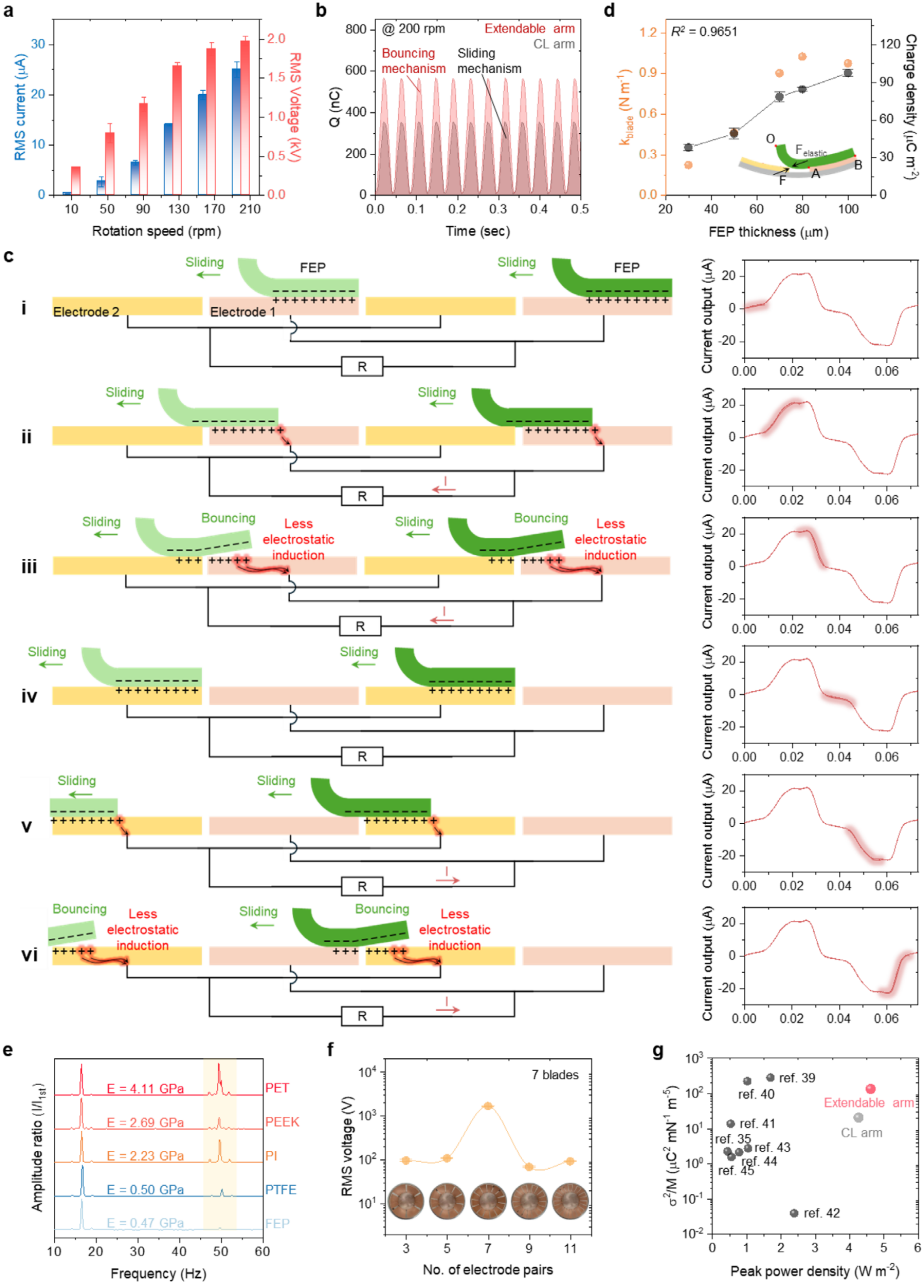
Parameters optimization of extendable swing arms. (a) The RMS current and voltage generated by the r‐TENG equipped with extendable swing arms with differing rotation speeds from 10 to 200 rpm. (b) The charge generated by the extendable (red) and CL (gray) swing arms. (c) Illustration of charge behaviors during sliding and bouncing motions, and their corresponding current waveform. (d) Spring constant of blades and corresponding charge density generated by the extendable swing arms as a function of blade thickness. (e) Bouncing behaviors comparison with varying elastic modulus. (f) Optimization of the number of electrode pairs. (g) The working condition‐independent energy conversion efficiency (*σ^2^/M*) and peak power densities for rotational TENGs in comparison with previous reports.

The output current, output voltage, and transferred charge of the r‐TENG with extendable swing arms as functions of the rotation speed are presented in Figure 2a and Figure S3a. We have chosen the root mean square (RMS) of the output values for a detailed numerical comparison and evaluation. The RMS output value, which is correlated with the area and frequency of the output peak, signifies the size of the output produced per unit time. The RMS for both current and voltage can be computed using these equations:

(1) $$I_{\mathrm{RMS}}=\sqrt{\frac{\int I^{2}\left(t\right)dt}{T}}\;-\sqrt{\frac{\int I_{noise}^{2}\left(t\right)dt}{T}}$$

(2) $$V_{\mathrm{RMS}}=\sqrt{\frac{\int V^{2}\left(t\right)dt}{T}}\;-\sqrt{\frac{\int V_{noise}^{2}\left(t\right)dt}{T}}$$

where *V(t)* and *I(t)* are the measured raw output voltages and currents, respectively. The *V_noise_(t)* and *I_noise_(t)* are the voltages and currents of the ambient electric noise, respectively, and *T* is the total measurement time. The current, voltage, and charge increase with increasing rotation speed due to more sufficient surface contact at a greater rotation speed, reaching 25.1 ± 1.5 µA, 1974.9 ± 58.5 V, and 505.1 ± 38.0 nC at the rotation speed of 200 rpm, respectively. Given that the short‐circuit charge of r‐TENGs relies on geometrical factors rather than rotation dynamics, i.e., the transferred charge is independent to the rotation speed (Figure S3b), such a bouncing is advantageous in terms of an additional charge‐feeding mechanism regardless of rotation speed while the rotor slides over the electrodes, as demonstrated by the average improved charge (*Q_bounce_* = 139.45 ± 14.98 nC) compared to the charge output from the sliding mechanism only (Figure 2b and Figure S3a). The working principle behind the additional charge‐feeding mechanism is schematically illustrated in tandem with the corresponding current output in Figure 2c. As the FEP blades slide over electrode 1, contact electrification occurs between the blades and the electrode, causing the surfaces of the blades to become negatively charged, as explained by the triboelectric series (Figure 2c (i)) [39]. While these charged blades come into contact with electrode 2 and sweep the electrode 2 surface, the electrostatic induction drives the electrons to move gradually from electrode 2 to electrode 1 (Figure 2c (ii)). Impinging the blades into electrode 2 edge induces a slight lifting of the blade tails, increasing the gap between the blades and electrode 1. This disruption in electrostatic equilibrium results in additional electrons transferring from electrode 2 to electrode 1 (Figure 2c (iii)). Such electron flows are depleted once the blades completely overlap with electrode 2 (Figure 2c (iv)). Subsequently, as the blades continue to slide and touch electrode 1 again, electrons flow back through the external circuit (Figure 2c (v)). The additional charge feeding mechanism also exists in the backflow (Figure 2c (vi)), slowing down current signal degradation while the blades cross electrodes and resulting in the square‐like waveform.

### 2.3 Importance of Blade Elasticity and Electrode Design

When the blades slide over the set of electrodes, the elasticity of the blades is crucial for the bouncing mechanism in r‐TENG, which determines both the contact area and the bouncing behavior. As each blade can be simplified as a flexible cantilever beam fixed at one end and free at the other, the elasticity of the blades can be quantified using the cantilever beam model. The spring constant (*k_blade_*) of the blade can be expressed as [40]

(3) $$k_{blade}=\frac{Ewt^{3}}{4l^{3}}$$

where *E* is the elastic modulus of the blade, and the geometric parameters, including *w*, *t*, and *l*, correspond to the width, thickness, and length of the blade, respectively. The blade can be divided into two sections (inset of Figure 2d): the part subjected to bending pressure from the shell (*l_OA_*), and the part in contact with the shell (*l_AB_*), so we set *l* = *l_OA_* in this study. The measured geometries and elastic modulus are detailed in Table S1. For a blade thickness of 30 µm, the *k_blade_* was found to be 0.22 N m^−1^ when subjected to a rotation speed of 140 rpm, and this value rises to 1.11 N m^−1^ when the blade has a thickness of 100 µm (Figure 2d). The contact area substantially decreases with increasing thickness due to the greater spring constant (Table S1). Interestingly, despite the reduced contact area, the corresponding charge density increased from 37.6 to 97.1 µC mm^−2^ with an increase in the *k_blade_*, exhibiting a high degree of correlation (*r^2^* = 0.97) (Figure 2d). We attribute this correlation to the greater tendency of the elastic blades with a higher spring constant to experience a strong restoring force as they sweep the depth profile of the stator inner wall, giving rise to significant blade bouncing. Further, we sought to vary the elasticity of the blades by replacing the blade materials, including polyethylene terephthalate (PET), polyetheretherketone (PEEK), polyimide (PI), and polytetrafluoroethylene (PTFE), at the given geometry. The bouncing behavior in the rigid polymer blades became remarkable due to the improved elastic modulus (Figure 2e). Although the charge densities appeared to be primarily influenced by the triboelectronegativity of the blades rather than the *k_blade_* (Figure S4), it is noteworthy that a larger *k_blade_* of FEP enhances the charge density by approximately 1.5 times compared to PTFE, given that FEP and PTFE show comparable triboelectric charge‐accepting characteristics [41], indicating that the high elasticity of blades is advantageous in terms of the bouncing mechanism in r‐TENG.

Subsequently, we investigated the impact of electrode architectures on the output performance of r‐TENG, specifically focusing on the number of electrode pairs and electrode thickness. Our findings reveal a significant increase in the RMS voltage of the r‐TENG, which escalates by an order of magnitude when the number of blades and electrode pairs is synchronized, as demonstrated in Figure 2f; Figure S5, and Table S2. Moreover, the RMS current and voltage attained maximum values of 17.7 µA and 1.8 kV, respectively, when the electrode thickness was set to 50 µm, as illustrated in Figure S6a. This observation underscores the importance of matching the extendable length of approximately 52.6 µm (Figure S6b) within a given geometric configuration to the electrode thickness, thereby facilitating synchronized bouncing behavior.

### 2.4 Power Output and Energy Conversion Efficiency

Figure S7 shows the RMS power behaviors with respect to the external load for r‐TENGs equipped with extendable arms and constant‐length arms at the rotation speed of 200 rpm. The RMS power density (*P_RMS_*) can be obtained by calculating the energy through the load in a unit area according to the following equation:

(4) $$P_{RMS}=\frac{{I_{RMS}}^{2}R}{A}$$

where *I_RMS_* is the RMS current, *R* is the resistance, and *A* is the contact area. The maximum RMS power density of 2.52 W m^−2^ was achieved for r‐TENG equipped with extendable arms at the external load of 70 MΩ, indicating a 27% improvement compared to the constant‐length (CL) arms. The energy conversion efficiency of r‐TENGs can be acquired by the following equation: [42]

(5) $$\eta =\frac{E_{2}}{E_{1}}=\frac{k_{2}S^{2}}{k_{1}nt}\;\times \frac{\sigma^{2}}{M}$$

where *E_1_* and *E_2_* are the input mechanical energy and the output electrical energy, respectively. The *k_1_* and *k_2_* indicate proportional coefficients for input and output energy, respectively. The *S*, *t*, σ, *n*, and *M* correspond to area, time, charge density, rotation speed, and torque, respectively. Compared with a calculation based solely on wind energy input and electrical energy output, this evaluation method takes into account blade drag, mechanical friction, and other structural losses involved in the actual energy conversion process, and therefore more accurately reflects the real energy conversion capability of the device. Notably, the ratio of the square of the charge density to the torque can be approximated as a measure of energy conversion efficiency that remains independent of the specific working conditions of the input energy. The measured torque, charge density, the square of charge density under unit torque (*σ^2^/M*), and peak power density are summarized in Table S3. Impressively, the *σ^2^/M* of 130.1 µC^2^ mN^−1^ m^−5^ and the peak power density of 4.6 W m^−2^ were achieved with the r‐TENG equipped with extendable arms at a rotation speed of 200 rpm. To our knowledge, our r‐TENG accomplishes remarkable power output alongside high energy conversion efficiency (Figure 2g), which is unprecedented in the literature to date [37, 42, 43, 44, 45, 46, 47, 48]. This impressive performance is likely due to the combinational sliding and bouncing motion of the elastic blades introduced by the extendable swing arms, which in turn provide the additional charge feeding mechanism and reduced frictional loss.

### 2.5 Wind Energy Harvesting with r‐TENG

**FIGURE 3:**
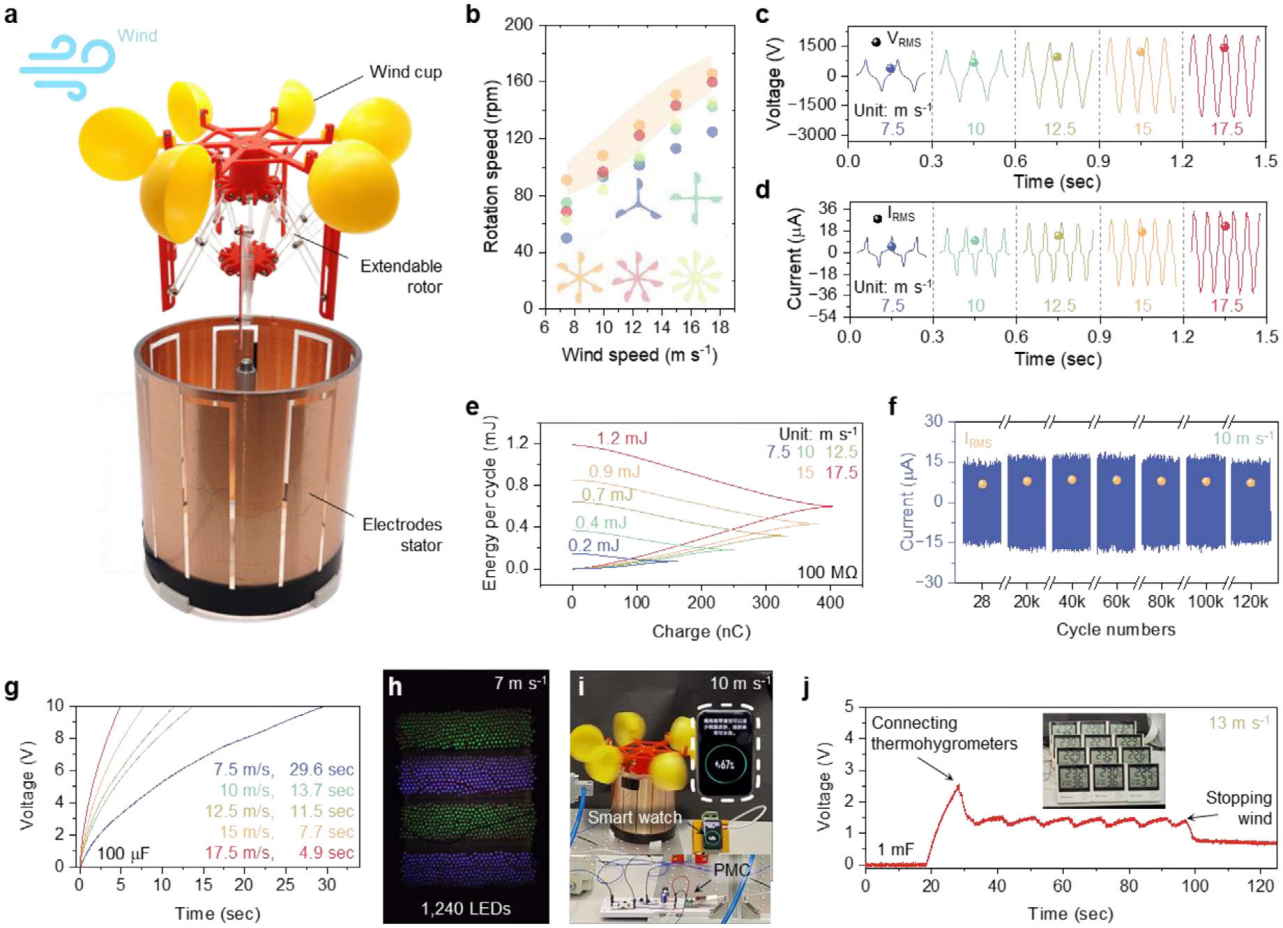
Wind energy harvesting capability of our r‐TENG. (a) Schematic of the r‐TENG featuring extendable swing arms and wind cups. (b) Optimization of the number of wind cups. (c,d) The waveforms representing voltage (c) and current (d) produced by our r‐TENG equipped with seven blades and six wind cups as a function of wind speed. (e) Energy–charge curve of our r‐TENG as a function of wind speed at an external load of 100 MΩ. (f) Durability of our r‐TENG in 120,000 cycles under the fresh breeze. (g) Charging 100 µF capacitor at different wind speeds. (h) Lighting up 1240 blue and green LEDs simultaneously at a wind speed of 7 m/s. (i) Supplying power for a smart watch by our r‐TENG at a wind speed of 10 m/s. (j) Twelve hygrothemometers in parallel, powered by our r‐TENG driven by a wind speed of 13 m/s. The inset indicates an optical image of 12 hygrothemometers working simultaneously.

After fine‐tuning the structural design parameters of the r‐TENG, we equipped it with wind blades to convert ambient energy into mechanical rotation and proceeded to its performance evaluation (Figure 3a). We first sought to optimize the number of wind blades, which determines the energy conversion efficiency of wind to rotational energy as well as the working window of wind speed [49]. As shown in Figure 3b, the r‐TENG achieved its maximum rotation speed when combined with a wind blade featuring six wind cups, across a wide range of wind speeds from 7.5 to 17.5 m/s, with a cut‐in speed of 5.7 m/s. It indicates the greatest energy conversion efficiency within a wind range of moderate breeze to a fresh gale, as defined in the Beaufort wind scale. This characteristic is particularly advantageous for ambient wind energy harvesting, given that the natural wind speeds of 4 to 9 m s^−1^ are most commonly observed [50, 51, 52]. A further increase in wind cups gives rise to the transition to turbulence in airflow behind the wind cups, lagging the rotation speed of r‐TENG. It is worth noting that the rotational speed of the r‐TENG increases with increasing wind speed. For the six‐wind‐cup configuration, a wind speed of approximately 21 m/s corresponds to a rotational speed of about 200 rpm. However, this wind condition falls within the strong gale regime and exceeds the practical operating range considered in this study. Therefore, device performance at higher wind speeds was not further investigated. Figure 3c, d explore the impact of varying wind speeds on the device's energy harvesting outputs. As wind speed increases, the device yields progressively higher output, peaking at an RMS voltage of ∼1425 V and an RMS current density of ∼1.4 mA m^−2^ at a wind speed of 17.5 m s^−1^. The results clearly indicate a linear correlation: higher wind speeds produce higher voltage (*R^2^* = 0.998) and current (*R^2^* = 0.992). Furthermore, the voltage, current, and transferred charge only slightly decreased as relative humidity (RH) rose from 20% to 60%, retaining over 70% of their initial values (Figure S8). This stability across typical moderate indoor RH levels (40%–60%) [53] valeted the practicality of our r‐TENG for indoor disinfection.

At a wind speed of 17.5 m s^−1^, the RMS voltage, current, and power of r‐TENG across the various external loads are presented in Figure S9. The r‐TENG achieves a maximum RMS power of 16.6 mW at an external load of 100 MΩ. Additionally, we measured the output energy generated per rotation at various wind speeds with an external load at 100 MΩ, achieved by synchronizing the timing of voltage (V) and charge (Q) measurements, as shown in Figure S10. The energy produced by a single electrode pair can be estimated using the following equation:

(6) $$E=\int_{0}^{t}VIdt=\int_{Q_{0}}^{Q_{t}}VdQ$$

By integrating the *V–Q* curve, we determined the total energy output from a single electrode pair. At a lower wind speed of 7.5 m s^−1^, this value was approximately 0.2 mJ, increasing to ∼ 1.2 mJ at a higher wind speed of 17.5 m s^−1^ (Figure 3e). Since each rotation of the r‐TENG engages 7 electrode pairs, the total energy generated per rotation at the highest wind speed is estimated to be ∼ 8.4 mJ, which is particularly high for a soft contact TENG. The durability of the r‐TENG was evaluated through a long‐term cycling test. As depicted in Figure 3f, the r‐TENG exhibited only minor fluctuations in its output current performance over the course of > 120,000 cycles, and the RMS current remained notably unchanged compared to the initial measurement after completing 120,000 cycles, demonstrating excellent stability and reliability. Correspondingly, FEP film after 120,000 cycles testing (Figure S11a) presented no obvious wear or structural damage in comparison to the initial sample (Figure S11b), confirming its mechanical integrity and supporting reliable electrical performance of r‐TENG.

Building on this, the r‐TENG was employed to charge the capacitors equipped with a power management circuit (Figure S12a). As wind speed increases, the charging rate gradually accelerates (Figure 3g), and the r‐TENG at a higher wind speed of 17.5 m s^−1^ can charge the capacitors of 100 µF up to 10 V in just 4.9 sec, indicating a charging rate of approximately 204 µC sec^−1^. Additionally, the r‐TENG under the wind speed of 10 m s^−1^ was able to charge a large capacitor of 1 mF up to 9.5 volts within 189 sec (Figure S12b). The wind‐driven r‐TENG can simultaneously light up 620 blue and 620 green light‐emitting diodes (LEDs) (Figure 3h and Movie S3). With the help of the power management circuit (PMC), the wind‐driven r‐TENG can be used as a power source for electronics, such as a smartwatch, a lamp, and a hygrothermometer. After ∼1.5 h of exposure to wind at a speed of 10 m s^−1^, the output current generated by the PMC reached the threshold of ∼ 100 µA (Figure S13), which is sufficient for charging a smartwatch. As a result of this power threshold being achieved, the user interface (UI) displayed on the smartwatch screen clearly indicated the current charging status (Figure 3i and Movie S4). Furthermore, the PMC facilitates continuous powering 30 W lamp (Movie S5) as well as 12 hygrothermometers (Figure 3j and Movie S6) at the wind speeds of 15 and 13 m s^−1^, respectively. Moreover, the r‐TENG can serve as a self‐powered wind speed sensor by virtue of the stable linear relationship between wind speed and electrical output. This enables real‐time, battery‐free wind velocity monitoring for intelligent ventilation, environmental monitoring, and smart building systems. Combined with its outstanding energy harvesting capability, the r‐TENG shows great promise for widespread applications in distributed IoT devices, portable electronics, self‐powered environmental sensors, and intelligent indoor air management systems.

### 2.6 Indoor Wind‐Driven Microbial Disinfection

**FIGURE 4:**
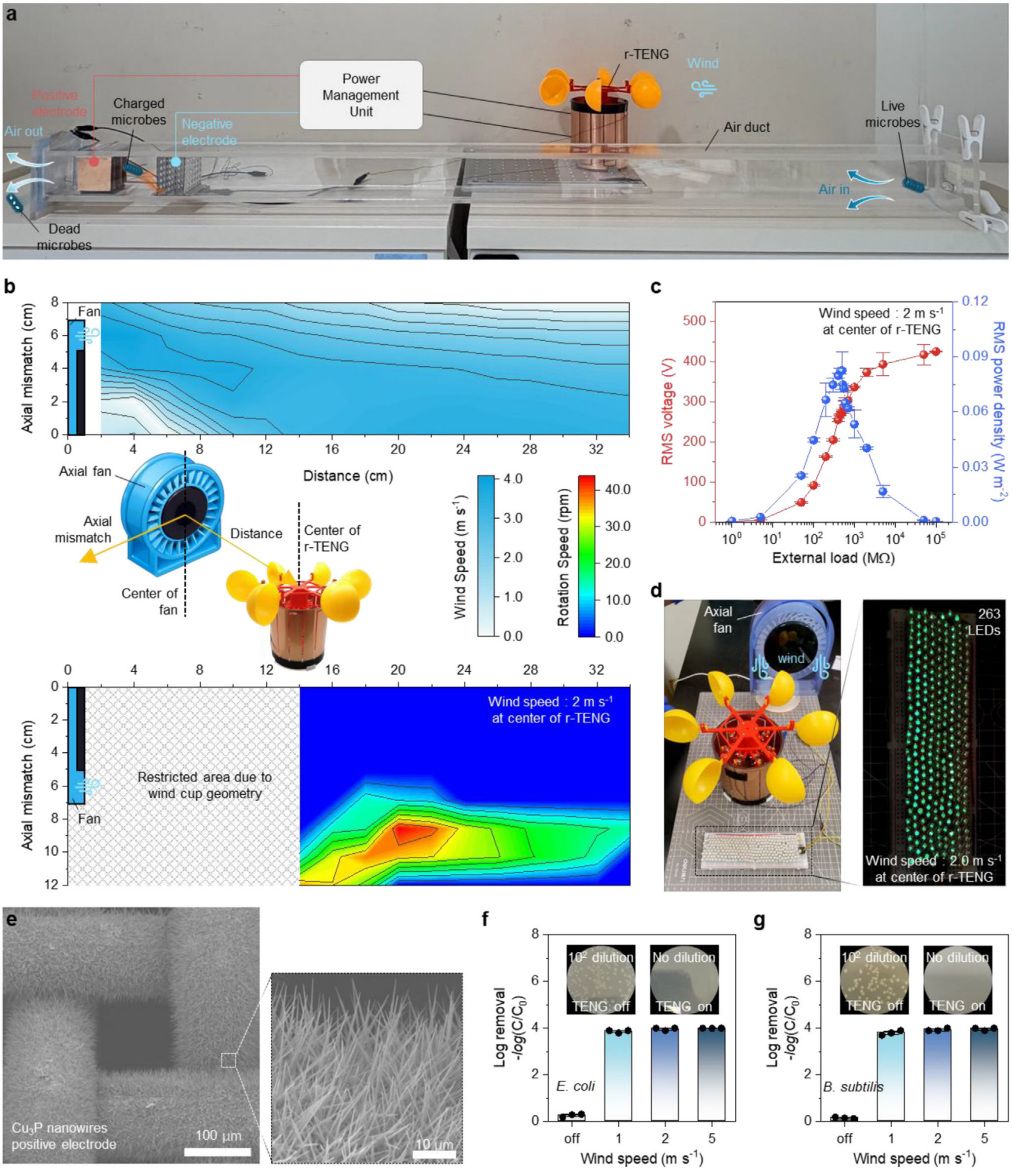
Indoor wind‐driven microbial disinfection. (a) Photograph of the indoor wind‐driven microbial disinfection platform, which comprises the wind‐driven r‐TENG energy harvester, a power management unit (PMU), and a three‐electrode disinfection filter positioned within a custom air duct. (b) Analysis of indoor airflow characteristics. Top: Measured wind speed contour map produced by an axial ventilation fan. Bottom: Measured rotation speed contour map of the axial fan‐driven r‐TENG, indicating a velocity of 2 m s^−1^ at the center of the device. (c) RMS voltage (red) and power density (blue) of axial fan‐driven r‐TENG as a function of external loads. (d) Demonstration of the power generation capability. (e) Scanning electron microscopy (SEM) images at low (left) and high (right) magnification, revealing the morphology of the vertically aligned Cu_3_P nanowires synthesized on the positive copper mesh electrode. (f, g) Antimicrobial efficacy of the self‐powered system against (f) Gram‐negative *E. coli* and (g) Gram‐positive *B. subtilis*. The data represent disinfection performance at varying wind speeds, while the insets display representative agar plates comparing bacterial colony growth with the r‐TENG output disconnected (TENG off) versus connected (TENG on).

Wind is often classified as an intermittent energy resource; however, artificially induced indoor wind, primarily used to mitigate cross‐infection of airborne pathogens, can serve as a stable, continuous energy source. To extend the utility of r‐TENG to indoor settings, we demonstrated an indoor wind‐driven microbial disinfection platform comprising an r‐TENG, a power management circuit with rectifiers, and a three‐electrode disinfection filter for airborne pathogen removal (Figure 4a). We have chosen a ventilation fan as an example of indoor wind sources, as it has been employed in a wide range of indoor spaces, including toilets, changing rooms, meeting rooms, and offices. Unlike blowers that generate high‐pressure, localized air streams (Figure S14), these fans typically employ an axial mechanism, producing lower‐pressure airflow with broader spatial coverage (Figure 4b, top). The r‐TENG attained a maximum rotational speed of 44 rpm under a wind speed of 2 m s^−1^ (Figure 4b, bottom), confirming the operational viability of r‐TENG even in low‐pressure aerodynamic conditions. Notably, the generator delivered a maximum RMS voltage of 426 V at velocities as low as 2 m s^−1^ (Figure 4c) and successfully powered approximately 260 light‐emitting diodes (LEDs) at 2 m s^−1^ (Figure 4d), suggesting that the capability of our r‐TENG to harvest sufficient energy from ambient indoor winds to drive the microbial disinfection system effectively [54, 55].

Building upon design parameters optimized in our previous works [54, 55], the three‐electrode disinfection filter incorporates a four‐layer aluminum macro‐mesh (5 mm square apertures) functioning as the cathode, paired with an anode composed of a copper plate modified with copper phosphide nanowires (Cu_3_P NWs). A copper ground electrode is integrated in parallel with the positive terminal to ensure electrical stability (Figure S15). The Cu_3_P NWs were fabricated via a scalable, two‐stage protocol [56]. Initially, copper hydroxide (Cu(OH)_2_) precursors were grown on a woven copper mesh (dimensions: 5 cm × 5 cm; 100 mesh) through electrochemical anodization. Subsequent phosphidation converted these structures into vertically aligned Cu_3_P NWs distributed uniformly across the substrate, measuring approximately ∼10 µm in length and ∼380 nm in diameter (Figure 4e). This chemical transformation was visually corroborated by a distinct chromatic shift from blue to black (Figure S16). During operation, bioaerosols are directed through a custom duct at a steady velocity of 1 m s^−1^, sequentially traversing the negative and positive/ground electrode array. To assess antimicrobial efficacy, we utilized *Escherichia coli* (*E. coli*) and *Bacillus subtilis* (*B. subtilis*) as representative Gram‐negative and Gram‐positive models, respectively. As illustrated in Figure 4f, g, the self‐powered microbial disinfection platform demonstrated robust sterilization capabilities driven by the indoor wind source, and complete inactivation was achieved for both *E. coli* (> 3.8 log reduction; > 99.98% efficiency) and *B. subtilis* (> 3.7 log reduction; > 99.98% efficiency) under fan speeds spanning 1 to 5 m s^−1^. In sharp contrast, disconnecting the r‐TENG output resulted in negligible bacterial removal (< 0.32 log reduction), highlighting the system's dependence on the generated electric field. Comparative agar plating assays (insets in Figure 4f, g) further visualize the stark difference in colony formation between the active r‐TENG treatment and the passive control groups at a wind speed of 2 m s^−1^. Post‐treatment morphological analysis via scanning electron microscopy (SEM) elucidated the underlying inactivation mechanism. Both bacterial strains exhibited distinctive surface lesions consistent with electroporation pores (∼100 nm) (Figures S17a, b). The presence of these structural defects confirms that irreversible electroporation, rather than chemical oxidation, ion toxicity, Joule heating, or mechanical trauma, serves as the dominant mode of sterilization in this system [54, 57].

## 3 Conclusion

In summary, this work establishes a robust framework for enhancing the performance and longevity of rotational triboelectric nanogenerators by strategically implementing extendable swing arms. By engineering a dynamic blade‐electrode interaction that leverages a combined sliding‐and‐bouncing motion, we successfully overcame the limitations of traditional frictional contacts, achieving superior charge‐transfer efficiency and minimizing mechanical wear. The optimized device not only exhibited long‐term stability exceeding 120,000 cycles but also demonstrated exceptional sensitivity to low‐grade kinetic sources, effectively harvesting energy from gentle indoor airflows. Beyond structural optimization, integrating our r‐TENG with a three‐electrode disinfection filter demonstrates a transformative application in building hygiene. The system's ability to induce lethal electroporation in both Gram‐negative and Gram‐positive bacteria, driven solely by ventilation‐induced wind, offers a compelling, chemical‐free alternative for mitigating airborne biological hazards. Consequently, this technology provides a scalable pathway toward self‐sustaining smart building infrastructure, where ambient waste energy is repurposed to ensure both operational autonomy for electronics and enhanced environmental safety.

Despite these promising results, several limitations remain. The present device still involves a relatively complex mechanical assembly, its output is sensitive to high‐humidity environments, and its structural design may require customization for different installation scenarios. In addition, although this work focuses on achieving high electrical output, reduced abrasive loss, and long‐term stability, the use of recycled triboelectric materials remains an important direction for improving overall sustainability. Accordingly, future work will focus on simplifying the fabrication process for scalable production, enhancing long‐term stability under harsh operating conditions, integrating the device with IoT nodes and self‐powered wind speed sensors, optimizing its modular design for smart‐building applications, and incorporating efficient power‐management strategies for broader practical deployment. The exploration of recycled and environmentally friendly materials in rotational TENG systems will also be considered in future designs. Overall, this study provides a promising route toward sustainable, self‐powered smart‐building technologies that couple ambient energy harvesting with environmental health functions.

## 4 Experimental Section

### 4.1 Fabrication of r‐TENG

The whole r‐TENG was measured 26 cm in length and width, and 17.6 cm in height. The stator chassis was constructed from a polycarbonate (PC) cylinder with an external diameter of 11 cm, a height of 12 cm, and a wall thickness of 3 mm, which was selected for its high mechanical strength and low surface friction. Centered coaxially within this shell was a machined steel driving shaft (10 mm diameter, 105 mm height), engineered with a stepped profile (6 mm diameter, 5 mm length) to accommodate two precision bearings (NSK MR106ZZ). The shaft featured a 1 mm chamfer at the superior end for ease of assembly and a 3 mm threaded aperture at the base for secure mounting. To form the triboelectric interface, fourteen rectangular copper electrodes (50 µm thickness, 21 mm width, 112.5 mm length) were uniformly adhered to the inner circumference of the PC substrate. These electrodes were electrically configured into two interdigitated sets by connecting non‐adjacent strips, resulting in a gap distance of 2.34 mm and an effective interaction length of 105 mm. The rotor assembly was designed to facilitate variable motion regulation via a vertical sliding mechanism. Key structural components—including the central hub, vertical slider, and blade holders—were fabricated via additive manufacturing (Prusa i3 MK3S+) using high‐strength and lightweight polylactic acid (Polymaker PolyLite PLA Pro). The hub was press‐fitted onto the upper shaft section, engaging the bearings to drive the slider's vertical displacement relative to the rotation. The extendable swing arms were constructed from laser‐cut high rigidity, smooth surface, and good wear resistance acrylic struts (8 mm width, 3 mm thickness, 98 mm length). Each strut featured three symmetrical 3 mm through‐holes spaced at 45 mm intervals, serving as hinge points for the hub, slider, and blade holder connections; these articulations were secured using 2.5 mm bolts and locknuts. Crucially, the blade holders were engineered with both circular and slotted apertures to enable the specific sliding‐and‐bouncing kinematics required for enhanced energy harvesting. Fluorinated ethylene propylene (FEP) films (70 µm thickness, 100 mm length, 26 mm width) served as the active triboelectric blades with strong electronegativity, high flexibility, and excellent durability, attached to the holders using double‐sided tape with a 5 mm overlap. A total of seven blade assemblies were distributed radially around the shaft at equispaced intervals of 51.43°. The device featured a modular coupling interface above the hub to accommodate different driving sources. For controlled laboratory characterization, a connector with a D‐profile aperture was utilized to interface with a stepper motor shaft. Conversely, for ambient energy harvesting, the system was fitted with a wind‐scavenging array consisting of six hemispherical cups (60 mm diameter) made of lightweight acrylonitrile butadiene styrene (ABS), enabling the device to start rotating at low wind speeds. These cups were arranged radially at 60° intervals with a rotational radius of 10 cm from the central axis to maximize torque generation from airflow.

### 4.2 Electrical and Optical Characterization

The rotation was driven by the commercial programmable permanent‐magnet brushed DC motor (Yahboom MD520Z19_12 V) and the controller (Yahboom AT8236), with rotation speed controlled by adjusting the input voltage. The wind was generated by compressed air, with the wind speed controlled by adjusting the output pressure with a precision regulator (SMC IR2020‐02BG). The axial ventilation fan (Ranvoo FZ1) was selected as an example of indoor wind sources. Wind speed was measured using an anemometer (Delixi DECEMDAH30). An oscilloscope (Agilent DSO‐X‐2012A) equipped with a preamplifier (SRS SR‐570) was utilized to measure the output voltage and current from the r‐TENG. The voltage and current of the r‐TENGs were measured with high‐impedance (100 MΩ) and low‐impedance (50 Ω) probes, respectively, without connecting external loads. A digital multimeter (Keithley 6514) was used to analyze the generated charge. The LEDs, lamp, smartwatch (Huawei Watch Fit 1), and hygrothermometers were directly connected to a power management circuit along with r‐TENGs. The rotational motions of blades were captured by a high‐speed camera (iX i‐SPEED 510). The surface morphology of the FEP film was observed by an optical microscope (AS ONE MT‐320).

### 4.3 Fabrication of Three‐Electrode Disinfection Filter

The negative electrode assembly was constructed from 0.5 mm thick aluminum foil (Alfa Aesar), precision‐cut into 6 cm × 6 cm squares. To form a macro‐mesh structure, square apertures (5 mm × 5 mm) were uniformly distributed across the surface. Three such macro‐mesh layers were subsequently mounted within an acrylic holder with a consistent interlayer spacing of 5 mm. The positive electrode, composed of copper phosphide nanowires on a copper substrate (Cu_3_P NW‐Cu), was synthesized via a two‐stage protocol. Initially, a rectangular copper foil (6 cm × 2 cm; Alfa Aesar, 0.5 mm thick) was surface‐treated with 1 M HCl (Sigma) and rinsed with deionized (DI) water to eliminate contaminants. This substrate underwent electrochemical anodization in a 3.0 m NaOH solution (Sigma) at a constant current density of 5 mA cm^−2^ for 30 min using a DC power supply (UNI‐T, UTP1303), yielding copper hydroxide nanowires (Cu(OH)_2_ NWs). In the subsequent phosphidation phase, the anodized sample was positioned in the downstream zone of a tube furnace (temperature maintained at 120°C), while a superstoichiometric amount of sodium hypophosphite (Sigma) was placed at the furnace center (300°C). Following a 15‐min argon purge, the reaction proceeded for 90 min, after which the system was allowed to cool naturally to ambient temperature under an argon atmosphere, resulting in the final Cu_3_P NW‐Cu electrode. The ground electrode consisted of a plain copper foil with dimensions identical to the positive electrode. The final filtration unit integrated three positive and three ground electrodes arranged in a parallel configuration with 1 cm spacing. Surface morphology was examined using scanning electron microscopy (SEM; JEOL, JSM‐7001F) at an accelerating voltage of 15 kV.

### 4.4 Wind‐Driven Disinfection System

The integrated electrode module comprising the macro‐mesh cathode and the anode/ground array was housed within a square acrylic duct (6 cm × 6 cm cross‐section). The electrodes were energized by the rectified output of the r‐TENG. To ensure the development of fully laminar airflow, the duct length was extended to 1.4 m. Bioaerosols were introduced into the system using a high‐performance air compressor nebulizer (Philips) loaded with a concentrated microbial feed solution. Airflow velocity within the duct was regulated at 1 m s^−1^ via compressed gas injection, while relative humidity was maintained at 30% using a secondary nebulizer generating a water mist. Environmental parameters, including flow rate, humidity, particle concentration, and pressure drop, were continuously monitored via integrated sensors.

### 4.5 Disinfection Performance Analysis

*Escherichia coli* (*E. coli*; ATCC 15597) and *Bacillus subtilis* (*B. subtilis*; ATCC 23857) were selected as model pathogens. Bacteria were cultured in Tryptic Soy Broth (TSB, Sigma) to the log phase (12 h), harvested via centrifugation at 1500 g (HITACHI, RX2 series), and washed three times with DI water. The cells were ultimately resuspended in DI water to achieve a feed concentration of approximately 10^8^ CFU mL^−1^. Following passage through the disinfection system, the bacteria‐laden airflow (total volume: 0.5 m^3^) was captured in a liquid impinger containing 500 mL of sterilized DI water. Microbial recovery was quantified using standard spread plating techniques [58, 59]. The disinfection efficiency was calculated using the log reduction value (LRV) according to Equation 7:

(7) $$Efficiency=-\log_{10}\left(C/C_{0}\right)$$

where *C* and *C*_0_ denote the microbial concentrations recovered with and without the operation of the disinfection system, respectively. This metric allows for the precise quantification of inactivation rates exceeding 90% even at high bacterial loads [60]. All samples were serially diluted and plated in triplicate, with incubation performed at 37°C for 12 h.

### 4.6 Bacterial Sample Preparation for SEM

For morphological analysis, bacterial specimens were isolated by centrifugation at 1500 g for 5 min at 15°C. Following supernatant removal, the pellets were subjected to overnight fixation at 4°C in a solution containing 0.1 M phosphate buffer (pH 7.3; Sigma) and 2% glutaraldehyde (Sigma). The samples were subsequently dehydrated through a graded ethanol series (50%, 70%, 90%, and 100%) and dried in 100% tert‐butanol (Sigma) via lyophilization (ilShin BioBase, TFD 8501). The prepared specimens were dispersed onto metal grids for SEM imaging (JEOL, JSM‐7610F).

## Author Contributions

D.‐M.S., S.‐W.K., Y.‐H.H., Y.L., J.Z., and I.‐Y.S. conceived the overall research goals and aims. Y.L. and J.Z. designed the experiments of r‐TENG. Y.L. fabricated devices and performed the characterization with help from J.Z. and E.K. D.‐M.S., Y.‐H.H., and Y.L. performed the data analysis and organized the results of r‐TENG parts. S.‐W.K., Y.‐J.K., and I.‐Y.S. designed the indoor wind‐driven microbial disinfection system. I.‐Y.S., D.‐J.K., and J.J. conducted the SEM measurements. I.‐Y.S. and J.‐M.J. fabricated systems and performed the characterization with help from J.‐H.L. S.‐W.K. J.‐H.L., and I.‐Y.S. performed the data analysis and organized the results of microbial disinfection parts. All the authors contributed to writing the manuscript.

## Conflicts of Interest

The authors declared no competing interests.

## Supporting information




**Supporting File**: advs75294‐sup‐0001‐MovieS1.mp4.

**Supporting File**: advs75294‐sup‐0002‐MovieS2.mp4.

**Supporting File**: advs75294‐sup‐0003‐MovieS3.mp4.

**Supporting File** advs75294‐sup‐0004‐MovieS4.mp4.

**Supporting File**: advs75294‐sup‐0005‐MovieS5.mp4.

**Supporting File**: advs75294‐sup‐0006‐MovieS6.mp4.

**Supporting File**: advs75294‐sup‐0007‐SuppMat.docx.

## Data Availability

The data that support the findings of this study are available from the corresponding author upon reasonable request.
