# Supplementary material for: Concurrently Achieving 4.6 W/M2 and 120,000 Cyclability Enabled by Extendable Swing Arms in Rotational Triboelectric Nanogenerator
Source: Adv Sci (Weinh). 2026 Apr 14;13(38):e75294. doi: 10.1002/advs.75294 (PMC13335101; doi:10.1002/advs.75294)
Supplement: Supplementary file 7 — Supporting File: advs75294‐sup‐0007‐SuppMat.docx. [file ADVS-13-e75294-s002.docx]

**Supporting Information**

**Concurrently Achieving 4.6 W/m^2^ and 120,000 Cyclability Enabled by Extendable Swing Arms in Rotational Triboelectric Nanogenerator**

Yihong Lin^1,†^, Jiaming Zhou^1,†^, In-Yong Suh^2,†^, Eunjong Kim^1^, Kin Chiu Yip^1^, Dae-jin Kim^2^, Jang-Mook Jeong^2^, Young-Jun Kim^2^, Jinyoung Jeon^2^, Ju-Hyuck Lee^3^, Yoon-Hwae Hwang^4,^*, Sang-Woo Kim^2,^*, Dong-Myeong Shin^1,^*

^1^ Department of Mechanical Engineering, The University of Hong Kong, Pokfulam Road, Hong Kong SAR, China

^2^ Department of Materials Science and Engineering, Center for Human-oriented Triboelectric Energy Harvesting, Yonsei University, Seoul 03722, Republic of Korea

^3^ Department of Energy Science and Engineering, Daegu Gyeongbuk Institute of Science and Technology (DGIST), Daegu 42988, Republic of Korea

^4^ School of Transdisciplinary Engineering & BK FOUR Nanoconvergence Technology Division, Pusan National University, Busan 46241, Republic of Korea

* E-mail: yhwang@pusan.ac.kr (Y.-H.H.), kimsw1@yonsei.ac.kr (S.-W.K.), dmshin@hku.hk (D.-M.S.)

^†^ These authors contributed equally to this work.

**Table of Contents**

Supplementary Figures 1 – 16··············································································2

Supplementary Tables 1 – 3················································································18

Caption for Supplementary Movies·······································································21

References····································································································22

**Supplementary Figures**

**
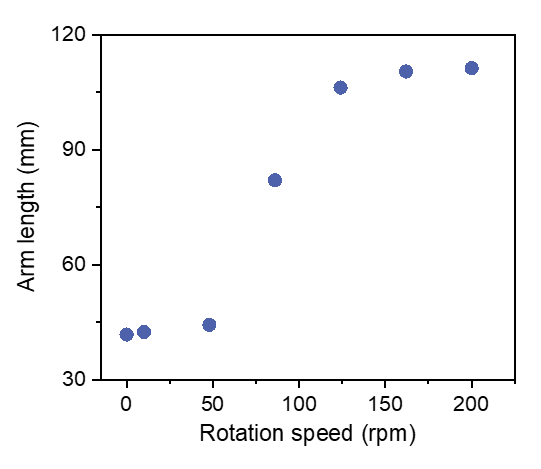
**

**Supplementary Figure 1.** The length of the extendable swing arm as a function of rotation speed.

**
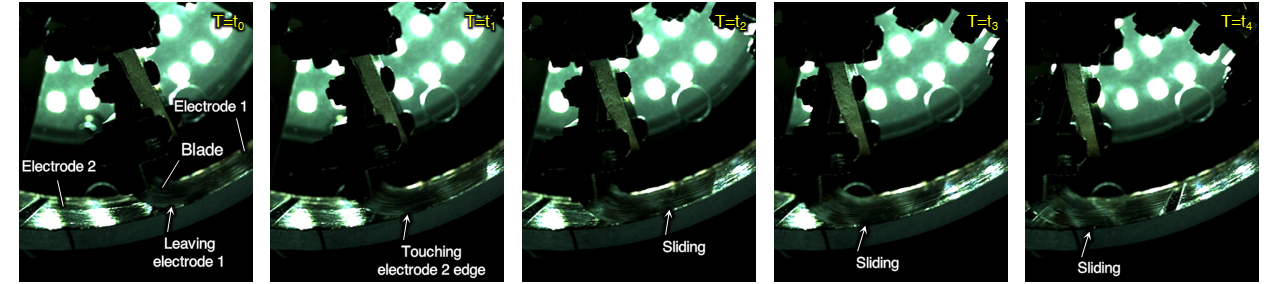
**

**Supplementary Figure 2.** Snapshot images of the sliding motion in the CL swing arm, captured by a high-speed camera.

**
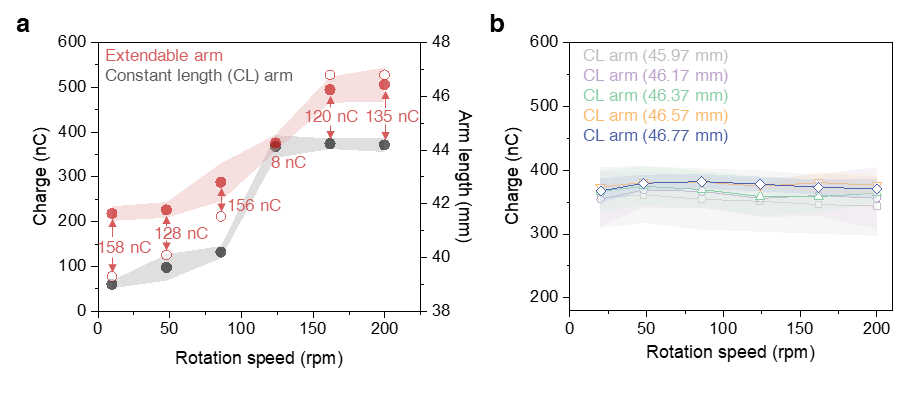
**

**Supplementary Figure 3. a**, The charge produced by the r-TENGs featuring extendable (red) and CL (grey) swing arms as a function of rotation speed, and their corresponding swing arm length (open symbol). **b**, The charge generated by the r-TENGs with differing CL arm lengths as a function of rotation speed. Output charges are independent of rotation speed at a given arm length.

**
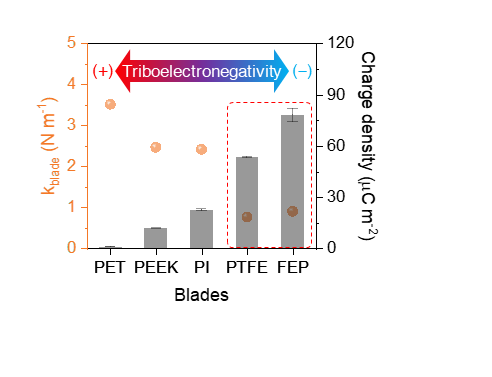
**

**Supplementary Figure 4.** Spring constant of blades and corresponding charge density generated by the extendable swing arms as a function of the blade materials. Although charge density was primarily determined by the triboelectronegativity of blades rather than by the spring constant, an increased spring constant nonetheless enhances charge density when the triboelectric charge-accepting properties are kept similar (highlighted with a red box).

**
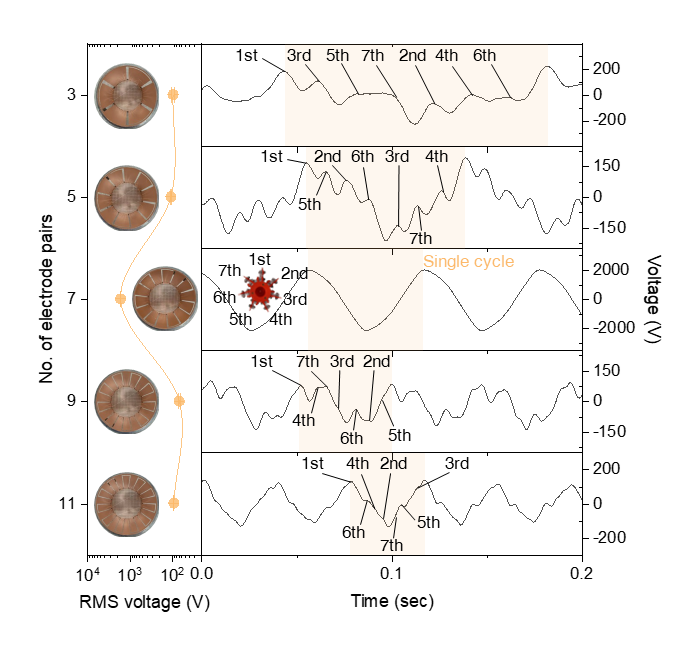
**

**Supplementary Figure 5.** The voltage waveforms of r-TENGs with varying the number of electrode pairs.

**
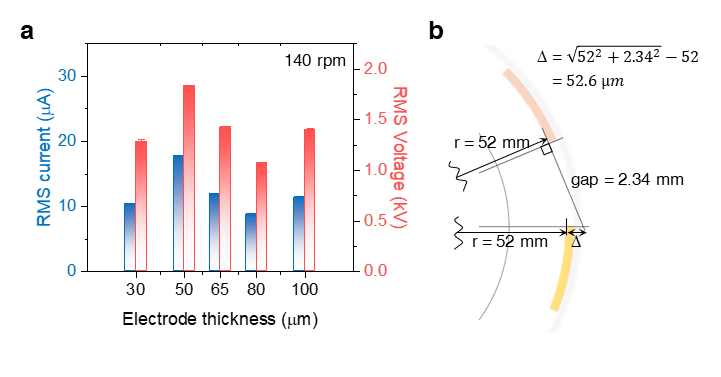
**

**Supplementary Figure 6. Optimization of electrode thickness. a,** The RMS current and voltage generated by the r-TENG equipped with extendable swing arms with differing electrode thickness from 30 to 100 μm. **b**, Geometric analysis of maximum extendable length during rotation at a given electrode gap.

**
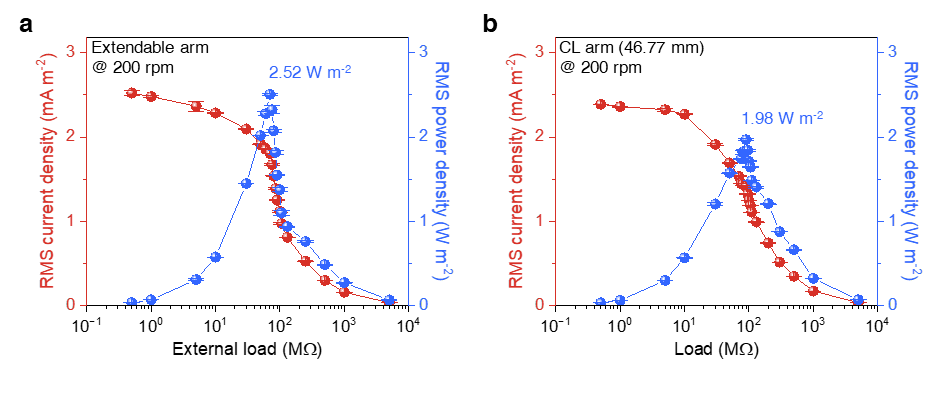
**

**Supplementary Figure 7. a**,**b**, The RMS current and power densities of r-TENG equipped with extendable (**a**) and CL (**b**) swing arms at a rotation speed of 200 rpm at various external loads from 0.5 to 5,000 MΩ.

**
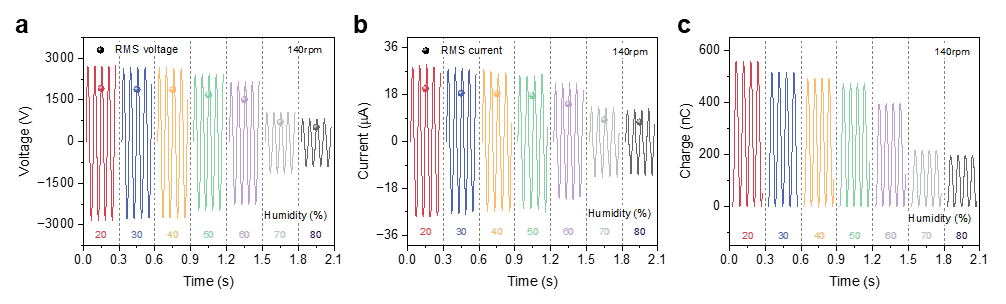
**

**Supplementary Figure 8.** Electrical performance of the r-TENG at 140 rpm under different relative humidity levels: (a) voltage, (b) current, and (c) transferred charge.

**
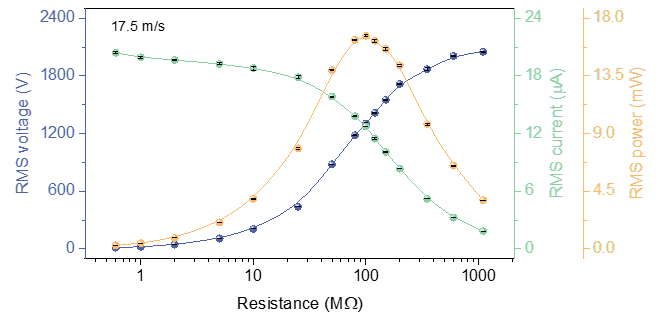
**

**Supplementary Figure 9.** The RMS voltage, current, and power of r-TENG equipped with extendable swing arms driven by a wind speed of 17.5 m/s at various external loads from 0.6 to 1,100 MΩ.

**
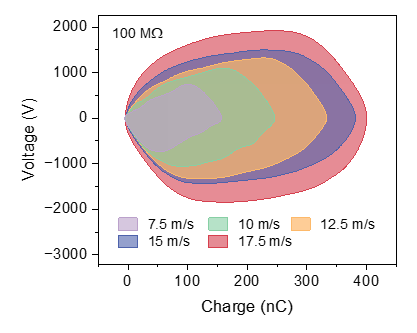
**

**Supplementary Figure 10.** Voltage–charge curves at different wind speeds at an external load of 100 MΩ.

**
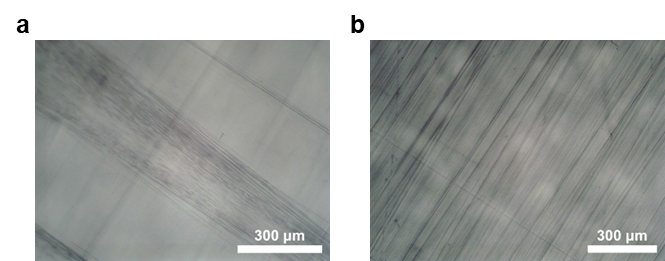
**

**Supplementary Figure 11.** Surface morphology of the FEP (a) after durability test and (b) before durability test.

**
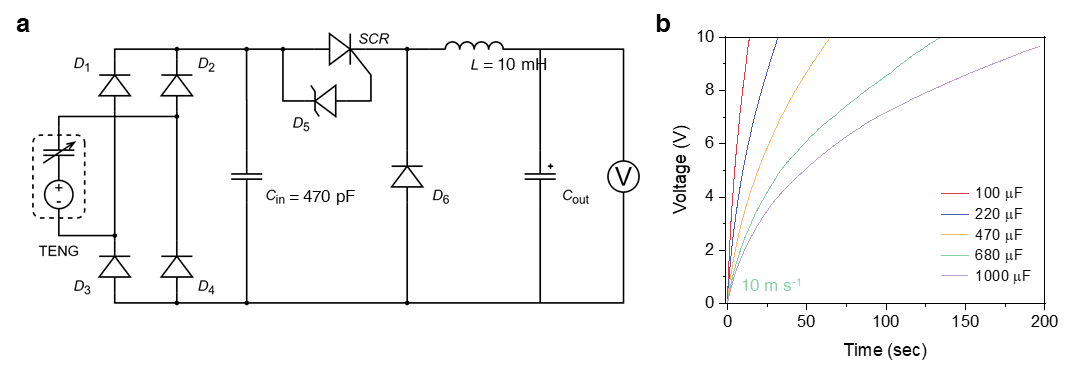
**

**Supplementary Figure 12. a**, Power management circuit diagram for charging the capacitors. **b**, Charging the various capacitors by our r-TENG at 10 m/s.

**
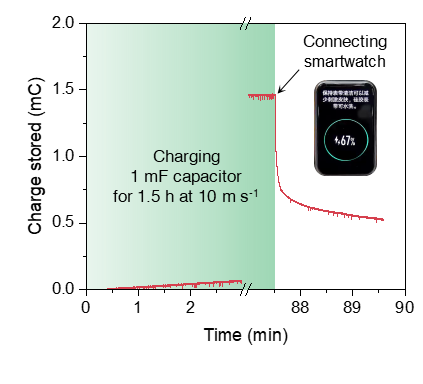
**

**Supplementary Figure 13.** Capacitor charging profile for powering a smartwatch.

**
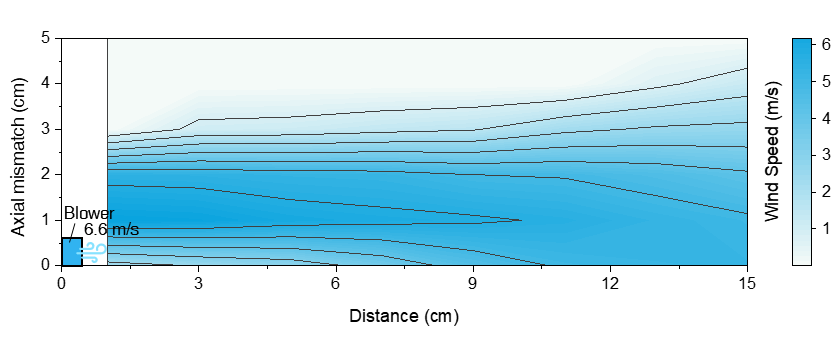
**

**Supplementary Figure 14.** Measured wind speed contour map produced by an air blower.

**
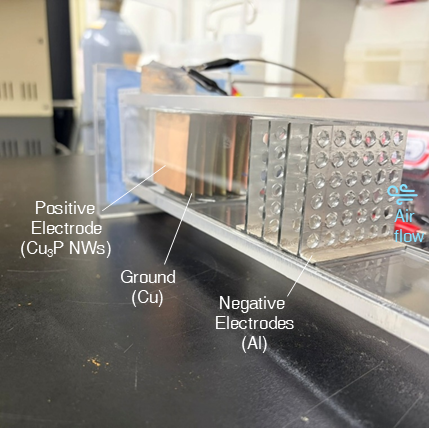
**

**Supplementary Figure 15.** Photograph of the three-electrode disinfection filter.

**
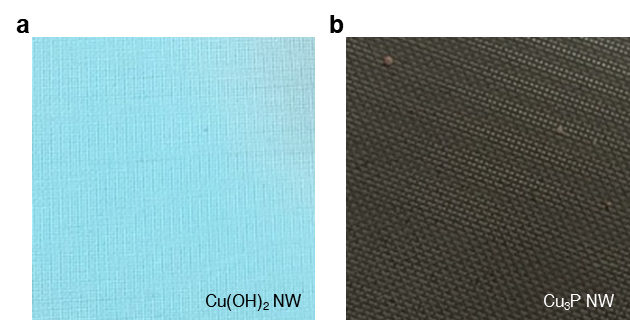
**

**Supplementary Figure 16.** Photographs of (a) Cu(OH)_2_ and (b) Cu_3_P NWs grown on Cu mesh.

**
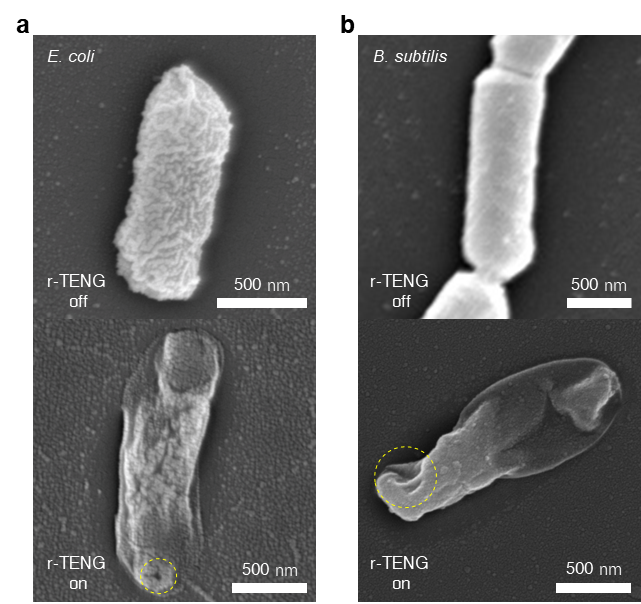
**

**Supplementary Figure 17.** SEM images of electroporated (a) *E. coli* and (b) *B. subtilis*.

**Supplementary Tables**

**Supplementary Table 1. Blade geometries and elastic modulus.**

| **Materials** | **Thickness (μm)** | **Width (mm)** | **l_OA_ (mm)** | **l_AB_ (mm)** | **Elastic modulus (GPa)** |
| --- | --- | --- | --- | --- | --- |
| FEP | 30 | 100 | 11.29 | 14.71 | 0.47 ± 0.01 |
|  | 50 |  | 14.72 | 11.28 |  |
|  | 70 |  | 16.48 | 9.52 |  |
|  | 80 |  | 18.05 | 7.95 |  |
|  | 100 |  | 22.94 | 3.06 |  |
| PET | 70 | 100 | 21.56 | 4.44 | 4.11 ± 0.09 |
| PEEK |  |  | 21.09 | 4.91 | 2.69 ± 0.17 |
| PI |  |  | 19.95 | 6.05 | 2.23 ± 0.07 |
| PTFE |  |  | 17.83 | 8.17 | 0.50 ± 0.03 |

**Supplementary Table 2. Time delay from the 1^st^ peak in the current waveforms.**

| **No. of Electrode pairs** | **Time delay from 1^st^ peak (ms)** | | | | | | |
| --- | --- | --- | --- | --- | --- | --- | --- |
|  | **Blades** | **2nd** | **3rd** | **4th** | **5th** | **6th** | **7th** |
| 3 | Actual | 78.26 | 17.32 | 98.10 | 38.28 | 116.98 | 56.60 |
|  | Theoretical | 81.63 | 20.41 | 102.04 | 40.82 | 122.45 | 61.22 |
| 5 | Actual | 21.04 | 48.08 | 70.80 | 9.96 | 32.62 | 58.30 |
|  | Theoretical | 24.49 | 48.98 | 73.47 | 12.25 | 36.74 | 61.22 |
| 9 | Actual | 35 | 19.5 | 9.00 | 42.42 | 28.89 | 12.78 |
|  | Theoretical | 34.01 | 20.41 | 6.80 | 40.82 | 27.21 | 13.61 |
| 11 | Actual | 15.96 | 34.7 | 12.32 | 26.18 | 8.30 | 23.48 |
|  | Theoretical | 16.70 | 33.40 | 11.13 | 27.83 | 5.57 | 22.26 |

**Supplementary Table 3. The measured torque, charge density, the square of charge density under unit torque (σ^2^/M), peak power density, and rotation speed.**

| **Author** | **Torque**  **(N·m)** | **Charge density**  **(μC·m^-2^)** | **σ^2^/M**  **(μC^2^·mN^-1^·m^-5^)** | **Peak power density***  **(W·m^-2^)** | **Rotation speed**  **(rpm)** |  |
| --- | --- | --- | --- | --- | --- | --- |
| This work  (Extendable arms) | 0.07245 | 97.09 | 130.1 | 4.63 | 200 | |
| This work  (Constant length arms) | 0.06885 | 36.98 | 19.9 | 4.27 | 200 | |
| Zhao *et al.*^[R1]^ | 0.176 | 16.3 | 1.5 | 0.56 | 200 | |
| Zhang *et al.*^[R2]^ | 0.5 | 32 | 2.0 | 0.79 | 200 | |
| Wang *et al.*^[R3]^ | 0.0825 | 13.4 | 2.2 | 0.44 | 1000 | |
| Han *et al.*^[R4]^ | 0.9 | 48.8 | 2.6 | 1.05 | ~68 | |
| Chen *et al.*^[R5]^ | 1 | 115.2 | 13.3 | 0.54 | ~200 | |
| He *et al.*^[R6]^ | 0.056 | 124 | 274.6 | 1.70 | 200 | |
| Li *et al.*^[R7]^ | 0.00297 | 25.4 | 217.2 | 1.03 | 220 | |
| Zhao *et al.*^[R8]^ | 0.175 | 2.56 | 0.0375 | 2.39 | 100 | |
| * We compared the peak power densities of the literature, as some articles do not mention the average power density. | | | | | |  |

**Supplementary Movies**

Supplementary Movie 1 | The high-speed camera video of rotating extendable swing arms.

Supplementary Movie 2 | The high-speed camera video of rotating constant length swing arms.

Supplementary Movie 3 | LEDs lighting powered by wind-driven r-TENG.

Supplementary Movie 4 | Smartwatch charging driven by r-TENG.

Supplementary Movie 5 | 30 W lamp powered by r-TENG.

Supplementary Movie 6 | Multiple hygrothermometers powered by r-TENG.

**References**

[R1] B. Zhao, Z. Li, X. Liao*, et al.*, "A Heaving Point Absorber-Based Ocean Wave Energy Convertor Hybridizing a Multilayered Soft-Brush Cylindrical Triboelectric Generator and an Electromagnetic Generator," *Nano Energy* 89 (2021): 106381.

[R2] C. Zhang, Y. Liu, B. Zhang*, et al.*, "Harvesting Wind Energy by a Triboelectric Nanogenerator for an Intelligent High-Speed Train System," *ACS Energy Letters* 6, no. 4 (2021): 1490-1499.

[R3] P. Wang, L. Pan, J. Wang*, et al.*, "An Ultra-Low-Friction Triboelectric–Electromagnetic Hybrid Nanogenerator for Rotation Energy Harvesting and Self-Powered Wind Speed Sensor," *ACS nano* 12, no. 9 (2018): 9433-9440.

[R4] J. Han, Y. Feng, P. Chen*, et al.*, "Wind-Driven Soft-Contact Rotary Triboelectric Nanogenerator Based on Rabbit Fur with High Performance and Durability for Smart Farming," *Advanced Functional Materials* 32, no. 2 (2022): 2108580.

[R5] P. Chen, J. An, S. Shu*, et al.*, "Super-Durable, Low-Wear, and High-Performance Fur-Brush Triboelectric Nanogenerator for Wind and Water Energy Harvesting for Smart Agriculture," *Advanced Energy Materials* 11, no. 9 (2021): 2003066.

[R6] L. He, C. Zhang, B. Zhang*, et al.*, "A High-Output Silk-Based Triboelectric Nanogenerator with Durability and Humidity Resistance," *Nano Energy* 108 (2023): 108244.

[R7] X. Li, Y. Cao, X. Yu*, et al.*, "Breeze-Driven Triboelectric Nanogenerator for Wind Energy Harvesting and Application in Smart Agriculture," *Applied Energy* 306 (2022): 117977.

[R8] T. Zhao, B. Niu, B. Liu*, et al.*, "A Rotating Tower-Like Triboelectric Nanogenerator for Ultrahigh Charge Density Breakthrough," *Nano Energy* 108 (2023): 108204.
